# Cerclage fixation without K-wires is associated with fewer complications and reoperations compared with tension band wiring in stable displaced olecranon fractures in elderly patients

**DOI:** 10.1007/s00402-021-04027-3

**Published:** 2021-07-08

**Authors:** Daniel Wenger, Gustav Cornefjord, Cecilia Rogmark

**Affiliations:** 1 Department of Orthopedics, Skåne University Hospital, Inga Marie Nilssons gata 22, 205 02 Malmo, Sweden; 2 Lund University, Lund, Sweden

**Keywords:** Olecranon, Fracture, Reoperation, Complication, Cerclage, Tension band wire

## Abstract

**Introduction:**

Tension band wiring of olecranon fractures has high reported rates of complications and reoperations. We aimed to compare classic tension band wiring to cerclage fixation without K-wires in the treatment of displaced olecranon fractures in elderly patients. The primary outcome was reoperation. Secondary outcomes included complications and patient reported outcomes. Outcomes following non-operative treatment were also studied.

**Materials and methods:**

Patients aged > 69 years presenting with Mayo class 2a and 2b olecranon fractures at our institution from 2004 through 2016 (*n* = 239) were eligible for study. Fracture type, treatment method, complications and reoperations were assessed from radiographs and hospital files. QuickDASH surveys were collected by mail.

**Results:**

Patients operated with tension band wire technique had more reoperations (*p* value 0.03): relative risk (RR) 2.2 (CI 1.08–4.3), odds ratio (OR) 2.6 (CI 1.05–6.4), and complications (*p* value 0.001): RR 2.5 (CI 1.51–4.1), OR 3.7 (CI 1.67–8.2), compared with those operated with cerclage technique. Non-operative treatment yielded similar complication (*p* value 0.2) and reoperation rates (*p* value 0.06) as cerclage fixation. The answer rate was insufficient to compare QuickDASH scores between treatments methods.

**Conclusions:**

In patients 70 years and older undergoing cerclage fixation for displaced stable olecranon fractures (Mayo class 2), the reoperation and complications rates were less than half of those in patients undergoing TBW fixation. Non-operative treatment yielded similar reoperation and complication rates to cerclage fixation, in selected cases.

**Level of evidence:**

III—retrospective comparative cohort study.

## Introduction

Olecranon fractures account for one fifth of all proximal fractures of the forearm, and are commonly caused by low energy trauma in elderly patients [1].

Undisplaced olecranon fractures, Mayo class 1a and 1b, can be treated non-operatively [2, 3]. Plate fixation is recommended for fractures with an unstable ulno-humeral joint (Mayo class 3a and 3b) [4].

Displaced but ulno-humerally stable olecranon fractures (Mayo class 2a and 2b) are commonly treated with either plate fixation or tension band wiring (TBW). TBW has high reported rates of local elbow complaints from surgical hardware, often necessitating reoperations [5–8]. Plate fixation, on the other hand, more frequently leads to serious complications compared with TBW [9]. A 2014 Cochrane review found no clear evidence favoring either method [10].

In light of the high complication risks with TBW and plate fixation, several studies suggest that non-operative treatment can be used also in class 2a and 2b fractures in elderly patients [11–14].

Problems with skin protrusion and local elbow issues can occur if the K-wires migrate [6, 15]. At our institution, cerclage fixation without K-wires has been an alternative to traditional TBW, since a previous study showed similar outcomes but a much lower reoperation frequency [7].

The aim of this study was to retrospectively compare cerclage fixation to TBW fixation with respect to reoperation rates (primary outcome), complication rates, and patient reported outcomes in elderly patients with Mayo class 2a and 2b olecranon fractures. Another secondary aim was to study outcomes following non-operative treatment.

## Materials and methods

All patients > 69 years of age presenting with olecranon fractures at our institution from 2004 through 2016 were eligible for study. Patients were identified from a local registry of in-hospital stays and outpatient visits using ICD-10-SE codes for olecranon fractures (S52.00 and S52.01) [16]. Fractures sustained before 2004 but operated during the study period were not included (*n* = 2).

**Fig. 1:**
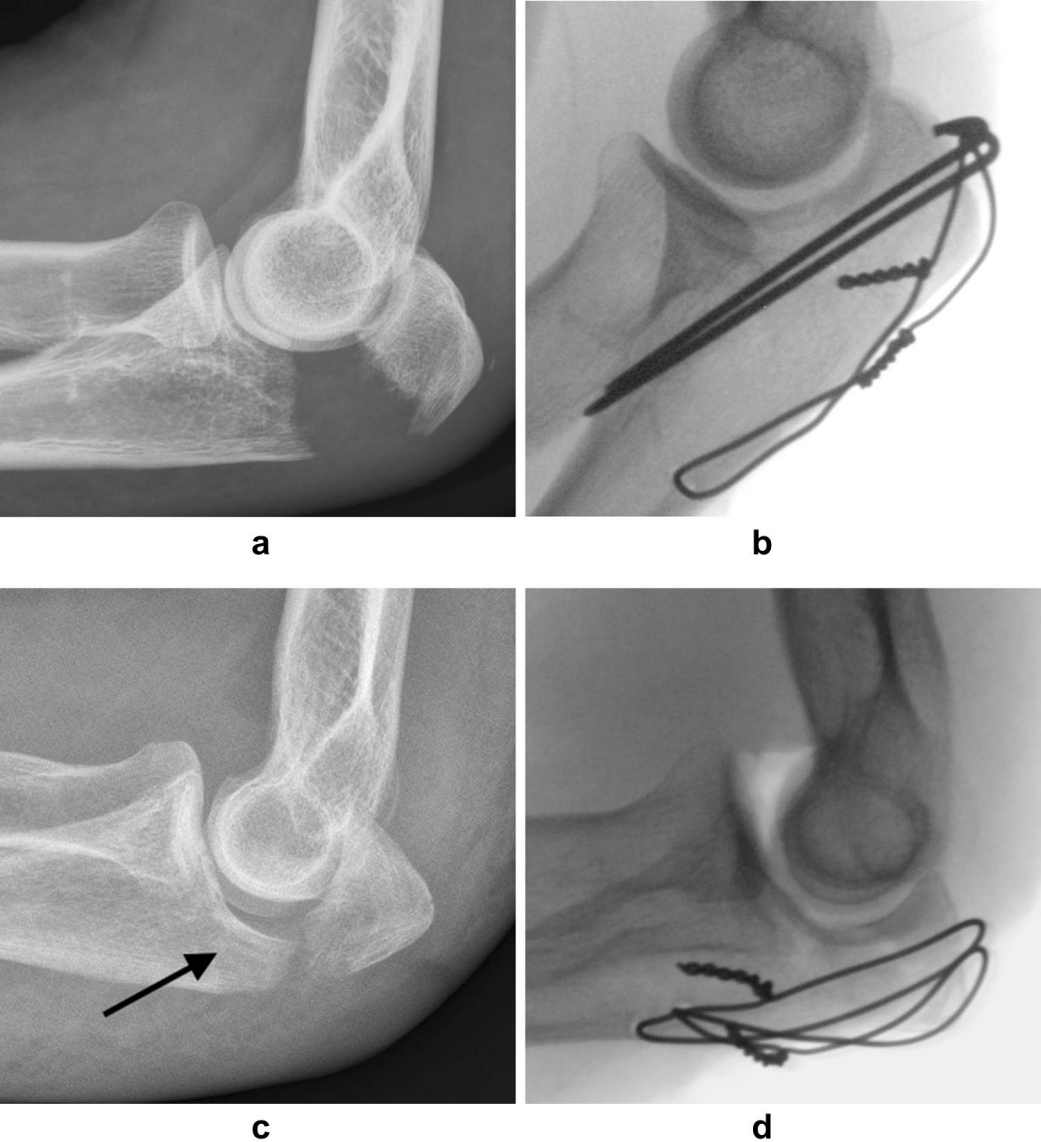
**a** Pre-operative lateral radiograph of a Mayo class 2a olecranon fracture. **b** Tension band wiring. Intraoperative lateral radiograph of the fracture in **a** after reduction and tension band wire fixation. The cerclage is proximally anchored in the bend of the K-wires. **c** Pre-operative lateral radiograph of a Mayo class 2b olecranon fracture. There is a depressed central fragment (arrow). **d** Cerclage fixation. Intraoperative lateral radiograph of the fracture in **c** after open reduction and fixation with two cerclages. The cerclages are in figure-8 and figure-0 configuration, respectively, and are proximally anchored in the triceps tendon

Fractures were described according to the Mayo classification [2]. Only Mayo class 2 fractures, with > 2 mm displacement but without ulno-humeral instability, were included. Subdivision was made into 2a (non-comminuted) and 2b (comminuted) (Fig. 1).

**Fig. 2:**
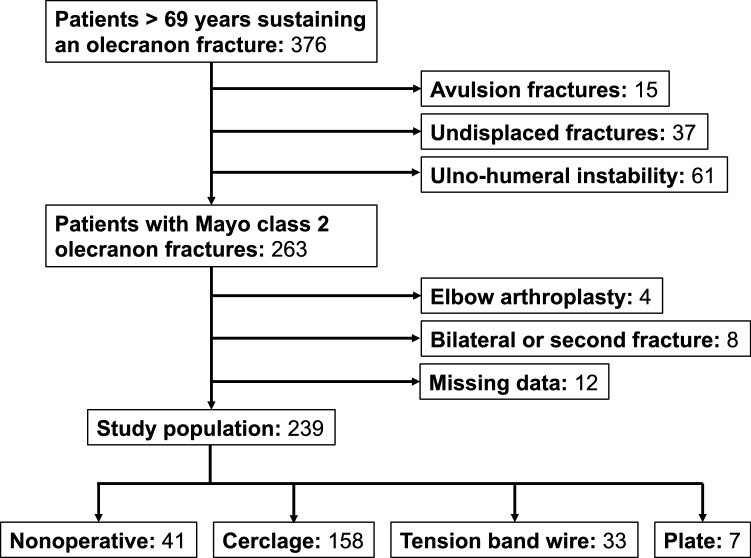
Exclusion flowchart

In total, 376 patients with olecranon fractures were identified, 263 of whom had Mayo class 2 fractures. After exclusion of 4 subjects with pre-existing elbow arthroplasty, 8 with bilateral fractures or a second ipsilateral fracture during the study period, and 12 with missing data, 239 subjects were included (Fig. 2).

Treatment methods were divided in TBW, cerclage fixation (using one or two cerclages), plate fixation (including combined plate and cerclage), and non-operative (Fig. 1). The primary aim of the study was to compare TBW with cerclage fixation with regard to unplanned return to theatre. Plate fixation is not standard practice for Mayo class 2 fractures, and non-operative treatment is typically reserved for elderly infirm patients at our clinic, introducing risk of selection bias in such cases. However, patients matching inclusion criteria and treated with these methods were also evaluated, both to give a complete view of treatment given in this age group, and to report patient related outcome after non-operative treatment compared to surgical treatment, which has not been done previously. Complications and patient reported outcomes were secondary study outcomes. The choice of treatment method was at the surgeon’s discretion. The post-operative protocol most commonly included 3 weeks in an elbow plaster cast, followed by active motion exercises, and gradually progressive weight bearing approximately from 6 weeks post-operatively. At the time of cast removal, patients met the treating physician (or if unavailable, another orthopaedic surgeon at the trauma service of the clinic), and a physiotherapist. In uneventful cases, rehabilitation was then given and followed by the physiotherapist. Post-operative radiographs were obtained if there was any clinical problem, or if the quality of intra-operative radiographs (often including stability provocation) was poor.

Data on reoperations, complications, and soft tissue integrity (open or closed fracture) were obtained from hospital files. A complication was defined as any complaint related to the fracture, leading to a health-care visit, or any deviation from the expected clinical course. Complications were classified as local elbow issues, sensory disturbances, severely impaired range of motion, infection or “other”. Infections were classified according to Dindo et al. not subdividing into A and B sublevels [17]. Reoperations were classified by underlying cause (local elbow issues, infection or “other”).

Patient-reported outcomes were evaluated using the QuickDASH score [18]. A difference of 12 points on the QuickDASH score was considered clinically important [19]. Questionnaires were sent by mail, together with a letter of consent, to the 107 subjects alive at follow-up.

The median time to follow-up was 5.2 years (IQR: 2.6–8.1) for surgically treated patients. In surgically treated patients who died during follow-up, the median time to death was 3.5 years (IQR: 1.8–6.4). The time of death was not available for non-surgically treated patients.

### Statistics

Comparisons of categorical variables were performed with the Chi-square test. The Mann–Whitney *U *test was used for group comparisons of continuous variables. Nonparametric tests were used as the continuous variables under investigation, age and QuickDASH, were not normally distributed (the Shapiro Wilk W-statistic yielded *p* values < 0.05). Data were presented as medians with interquartile range (IQR), as it was not normally distributed. Absolute risks with 95% confidence intervals (CI) were calculated using binomial distribution (exact method). Relative risks and numbers needed to harm with 95% confidence intervals (CI) were calculated according to Altman [20, 21].

For all analyses, an alpha level of 0.05 was considered statistically significant. Statistical analyses were performed using SPSS statistics for Macintosh (Armonk; NY; IBM corp.).

## Results

In the 239 included cases, the male to female ratio was 1:4.5. There were nine (4%) open fractures. Cerclage wiring was the most frequently used treatment method (*n* = 158), followed by non-operative treatment (*n* = 41), TBW (*n* = 33) and plate fixation (*n* = 7). A total of 73 surgeons performed 189 of the 198 operations (in 9 cases: 5 TBW and 4 plates, information was missing). Eleven surgeons performed five or more operations each.

In 32 (13%) cases, unplanned secondary surgery was performed (Table 1). The reoperation rate was not significantly affected by sex, or whether the fracture was comminuted or open. Patients undergoing reoperation were slightly younger (median age 78 vs. 82 years, *p* value 0.02). Patients treated with cerclage were less likely to undergo reoperation (*p* value 0.03): 13% (20 of 158), compared with TBW: 27% (9 of 33). The relative risk of undergoing secondary surgery was 2.2 (1.08–4.3) after TBW compared to cerclage fixation. The number needed to harm was 6.8 (3.6–76). Differences in reoperation rates were not statistically significant comparing cerclage fixation to non-operative treatment, or plate fixation.

**Table 1:** Patients with no secondary surgery compared with patients who underwent secondary surgery

	Total	No secondary surgery	Secondary surgery	*p* value	Relative risk (95% CI)^a^
Female	196	171 (87%)	25 (13%)	0.5	–
Male	43	36 (84%)	7 (16%)		–
Age, years^b^	–	82 (77–88)	78 (73–84)	0.02	–
Mayo 2a	139	118 (85%)	21 (15%)	0.4	–
Mayo 2b	100	89 (89%)	11 (11%)		–
Open fracture	9	9 (100%)	0	0.2	–
Non-operative	41	40 (98%)	1 (2%)	0.06^c^	0.19 (0.03–1.39)
Cerclage wire^d^	158	138 (87%)	20 (13%)	–	–
Tension band wire	33	24 (73%)	9 (27%)	0.03^c^	2.2 (1.08–4.3)
Plate	7	5 (71%)	2 (29%)	0.2^c^	2.3 (0.65–7.8)

Patients returning to theatre were more likely to have been operated with tension band wiring compared with those with cerclage fixation. Data given as frequency (%) unless otherwise stated

^a^Relative risk (95% CI) from comparison with cerclage fixation

^b^Data given as median (interquartile range)

^c^*p* value from comparison with cerclage fixation

^d^Of which 85 cases were operated with 1 cerclage, and 73 cases with 2 cerclages

A total of 52 (22%) patients had complications (Table 2). The complication rate was not significantly affected by sex, age, or whether the fracture was comminuted, or open. Patients treated with cerclage had an 18% (29 of 158) complication rate, which was lower than TBW (*p* value 0.001): 45% (15 of 33), relative risk 2.5 (1.51–4.1), or plate fixation (*p* value 0.04): 57% (4 of 7), relative risk 3.1 (1.51–6.4), and similar to non-operative treatment: 9.8% (4 of 41), relative risk 0.53 (0.20–1.43). The number needed to harm for TBW, compared to cerclage fixation, was 3.7 (2.4–8.5). Local soft-tissue problems were the most common complication, and the most common cause for reoperation (Table 3). Patients in the TBW group had local issues more often than those in the cerclage group (*p* value 0.01), and had a tripled risk of undergoing reoperation for this cause (*p* value 0.007). There were five cases of pseudarthrosis (3.2%) in the cerclage group, including one early secondary displacement and one occurring after implant removal due to infection, and one pseudarthrosis (3.0%) occurring after implant removal due to infection in the TBW group.

**Table 2:** Patients with uneventful healing compared with patients with complications

	Total	Uneventful healing	Complication	*p* value	Relative risk (95% CI)^a^
Female	196	153 (78%)	43 (22%)	0.9	–
Male	43	34 (79%)	9 (21%)		–
Age, years^b^	–	82 (77–88)	80 (75–86)	0.09	–
Mayo 2a	139	112 (81%)	27 (19%)	0.3	–
Mayo 2b	100	75 (75%)	25 (25%)		–
Open fracture	9	7 (78%)	2 (22%)	1	–
Non-operative	41	37 (90%)	4 (10%)	0.2^c^	0.53 (0.20–1.43)
Cerclage^d^	158	129 (82%)	29 (18%)	–	–
Tension band wire	33	18 (55%)	15 (45%)	0.001^c^	2.5 (1.51–4.1)
Plate	7	3 (43%)	4 (57%)	0.04^c^	3.1 (1.51–6.4)

Patients with complications were more likely to have been operated with tension band wiring or plate fixation, compared with those with cerclage fixation. Data given as frequency (%) unless otherwise stated

^a^Relative risk (95% CI) from comparison with cerclage fixation

^b^Data given as median (interquartile range)

^c^*p *value from comparison with cerclage fixation

^d^Of which 85 cases were operated with 1 cerclage, and 73 cases with 2 cerclages

**Table 3:** Comparison of treatment methods

	Non-operative	Cerclage	Tension band wire	Plate
Age, years^a^	88 (83–90)	81 (77–86)	77 (75–82)	78 (70–88)
Mayo class, 2b/2a	20/21 (49%)	63/95 (40%)	13/20 (39%)	4/3 (57%)
Open fracture	1 (2%)	8 (5%)	0	0
Reoperation due to infection	1 (2%)	4 (3%)	2 (6%)	2 (29%)
Reoperation due to local issues	0	13 (8%)	8 (24%)	0
Reoperation due to other causes	0	3 (2%)^b^	0	0
Local issues	0	17 (11%)	9 (27%)	0
Sensory deficits	1 (2%)	0	1 (3%)	0
Infections	3 (7%)	12 (8%)	4 (12%)	2 (29%)
Other complications	1 (2%)	12 (8%)	5 (15%)	1 (14%)
Complications, patients^c^	4 (10%)	29 (18%)	15 (45%)	4 (57%)
Secondary surgery, patients^d^	1 (2%)	20 (13%)	9 (27%)	2 (29%)

Patient characteristics, complications, and outcomes in 239 Mayo class 2a and 2b fractures. Data given as frequency (% within method) unless otherwise stated

^a^Data given as median (interquartile range)

^b^Other causes for reoperation include two cases of pseudarthrosis, and one case of posttraumatic osteoarthritis treated with total elbow arthroplasty

^c^*p *values for comparisons between methods are given in Table 2

^d^*p* values for comparisons between methods are given in Table 1

Complications and reoperations are listed in Table 3. There were 21 infections in total, Dindo class 1–4: 11, two, seven, and one, respectively. The patient with a class 4 complication developed sepsis with endocarditis, secondary to a wound infection. The infection rate did not significantly differ between the TBW group: 12%, and the cerclage group: 8% (*p* value 0.7).

There was a large spread in QuickDASH scores, with inadequate sample size (*n* = 41) to demonstrate any difference in patient reported outcome between treatment methods (28 responses from the cerclage group, 7 from the TBW group, and 3 from non-operatively treated and plate fixated, respectively).

## Discussion

In this cohort of elderly patients, cerclage fixation without K-wires was associated with fewer reoperations and complications compared with classical TBW. In an experimental setting, both fixation methods result in similar load-to-failure resistance [22]. The only previous clinical study comparing cerclage fixation to TBW also found a roughly doubled risk of reoperation with TBW, although the absolute risks were three times higher compared to our findings [7]. That investigation was performed at the same department, and we believe our results to better reflect the current situation with a higher threshold to hardware removal. A small case series of cerclage fixation without K-wires reported hardware removal in 4 of 17 patients, and good clinical outcomes at almost 5-year follow-up [23]. Another paper describing a similar technique with non-absorbable sutures also reported a low reoperation rate, although only 17 fractures and 11 osteotomies were included and the follow-up time was not specified [24]. Studies on TBW in olecranon fractures report reoperation rates between 22 and 82%, and complication rates between 23 and 63% [5, 6, 8, 9, 25–27].

Our finding of a lower all-cause complication rate in patients treated with cerclage (18%), compared with TBW (45%), has not been previously reported to our knowledge. The most common complication was local soft-tissue problems, and this occurred more often in the TBW group. Soft-tissue issues was also the main driver behind the high reoperation frequency in the TBW group, and behind the difference found between the study groups. K-wires can migrate and protrude under or through the skin [15]. In this study, we did not specifically assess the rate of K-wire migration.

The low number of patients with plate fixation in this cohort limits our ability to draw conclusions, even though the risk of complication or reoperation was three times higher following plating compared with cerclage fixation. At our institution, plate fixation is mainly used for unstable olecranon fractures (Mayo 3a and 3b). A prospective randomised trial comparing plate fixation to TBW reported lower complication rates in general (38 vs. 63%), but a higher frequency of serious complications in the plate group [9]. A previous meta-analysis, mainly including retrospective studies, also found a lower overall complication risk with plating compared with TBW [28].

In contrast to plate fixation and TBW, non-operative treatment yielded similar rates of complications and reoperations to cerclage fixation. However, the indication for non-operative treatment of a displaced olecranon fracture at our institution was a patient deemed too fragile to undergo surgery or patient refusal, introducing selection bias, including bias against secondary surgery. In this elderly cohort, 41 (17%) of 239 patients were deemed too frail for anaesthesia and surgery, or refused such treatment. That group of patients obviously differ from those who were operated, which is evident also from their median age of 88 years, so comparisons between them and surgically treated patients have to be made with caution. Previous studies, including one prospective randomised trial, have indicated that non-operative treatment can be a valid option for elderly patients with displaced, but stable, olecranon fractures [11–14]. Such patients often seem to accept the functional outcome of a non-union, which affects four out of every five patients who are treated non-operatively [12, 13, 29].

We included the validated upper extremity outcome measure QuickDASH in an effort to study patients’ perceptions, which may ultimately be the most relevant outcome. In this elderly cohort, where a significant proportion of the patients died before follow-up, the low response rate limited the analysis of differences in QuickDASH scores between treatment methods. Nevertheless, patient-reported outcomes and satisfaction are important measures that should be included in future studies. They should ideally be collected both at short-term and long-term follow-up.

There is a possibility of selection bias in the study groups, which limits the strength of our conclusions. Still, as patients treated with cerclage and TBW did not differ with regard to age, sex, Mayo class, or soft-tissue integrity, we believe that our main results most likely reflect differences between the two surgical methods. We believe the main determinant of treatment choice in this cohort was surgeon preference to, or familiarity with, either method. It is also possible that our results are not generalisable to some subgroup of patients. Our data could be used for calculating adequate sample sizes for hypothesis testing studies, with a higher level of evidence which is needed to give solid evidence-based treatment recommendations.

We did not have any reliable data on smoking status, a known risk factor for reoperations and complications. Tobacco use should preferably be controlled for in any prospective clinical trial of surgical treatments [30]. It is possible that some study participant was treated for a complication elsewhere which could have been missed, but as our department serves a geographical catchment area with no other hospital treating fractures (for primary treatment), and is the referral center (including the orthoplastic service) for orthopaedic surgery for all bordering geographic areas, we think it is unlikely that a significant number of such missed complications could have occurred. One should also be careful about generalizing our findings to younger patients, as poor bone quality in the elderly may increase the risk of K-wire migration.

### Conclusions

In patients 70 years and older undergoing cerclage fixation for displaced stable olecranon fractures (Mayo class 2), the reoperation and complications rates were less than half of those in patients undergoing TBW fixation. Non-operative treatment yielded similar reoperation and complication rates to cerclage fixation, in selected cases.

## Data Availability

Study data can be made available upon reasonable request to the corresponding author.
